# The Merits of Malaria Diagnostics during an Ebola Virus Disease Outbreak

**DOI:** 10.3201/eid2202.151656

**Published:** 2016-02

**Authors:** Emmie de Wit, Darryl Falzarano, Clayton Onyango, Kyle Rosenke, Andrea Marzi, Melvin Ochieng, Bonventure Juma, Robert J. Fischer, Joseph B. Prescott, David Safronetz, Victor Omballa, Collins Owuor, Thomas Hoenen, Allison Groseth, Neeltje van Doremalen, Galina Zemtsova, Joshua Self, Trenton Bushmaker, Kristin McNally, Thomas Rowe, Shannon L. Emery, Friederike Feldmann, Brandi Williamson, Tolbert G. Nyenswah, Allen Grolla, James E. Strong, Gary Kobinger, Ute Stroeher, Mark Rayfield, Fatorma K. Bolay, Kathryn C. Zoon, Jorgen Stassijns, Livia Tampellini, Martin de Smet, Stuart T. Nichol, Barry Fields, Armand Sprecher, Heinz Feldmann, Moses Massaquoi, Vincent J. Munster

**Affiliations:** National Institutes of Health, Hamilton, Montana, USA (E. de Wit, D. Falzarano, K. Rosenke, A. Marzi, R.J. Fischer, J.B. Prescott, D. Safronetz, T. Hoenen, A. Groseth, N. van Doremalen, T. Bushmaker, K. McNally, F. Feldmann, B. Williamson, H. Feldmann, V.J. Munster);; Centers for Disease Control and Prevention, Nairobi, Kenya (C. Onyango, B. Juma, B. Fields);; Kenya Medical Research Institute, Nairobi (M. Ochieng, V. Omballa, C. Owuor);; Centers for Disease Control and Prevention, Atlanta, Georgia, USA (G. Zemtsova, J. Self, T. Rowe, S.L. Emery, U. Stroeher, M. Rayfield, S.T. Nichol);; Ministry of Health and Social Welfare, Monrovia, Liberia (T.G. Nyenswah, M. Massaquoi);; Public Health Agency of Canada, Winnipeg, Manitoba, Canada (A. Grolla, J.E. Strong, G. Kobinger);; Liberian Institute for Biomedical Research, Charlesville, Liberia (F.K. Bolay);; National Institutes of Health, Bethesda, Maryland, USA (K.C. Zoon);; Médecins Sans Frontières, Brussels, Belgium (J. Stassijns, L. Tampellini, M. de Smet, A. Sprecher)

**Keywords:** Ebola, disease outbreak, malaria, Plasmodium, diagnostics, PCR, West Africa, public health, Guinea, Liberia, Sierra Leone, Ebola virus disease, viruses, Ebola virus

## Abstract

Malaria is a major public health concern in the countries affected by the Ebola virus disease epidemic in West Africa. We determined the feasibility of using molecular malaria diagnostics during an Ebola virus disease outbreak and report the incidence of *Plasmodium* spp. parasitemia in persons with suspected Ebola virus infection.

The Ebola virus disease (EVD) epidemic occurring in West Africa is unprecedented in its duration and scale; as of October 28, 2015, a total of 28,575 suspected, probable, and confirmed cases, including 11,313 deaths, have been reported (*1*). Healthcare workers have been severely affected, and the epidemic has resulted in an almost complete breakdown of the public health infrastructure that undoubtedly resulted in many deaths from otherwise treatable conditions and diseases. Particularly concerning are the effects of lapses in childhood vaccination, antenatal and emergency obstetric care, HIV treatment, and malaria control (*2*–*7*).

Malaria is a major public health concern in Guinea, Liberia, and Sierra Leone, the 3 countries most affected by the EVD epidemic. In 2012, these countries accounted for ≈9 million malaria cases and 30,566 associated deaths (*8*). Symptoms of Ebola virus (EBOV) infection and malaria overlap to a great extent, especially early during the course of disease; fever, headache, chills, and vomiting are observed frequently in both diseases. Malaria transmission occurs year-round in Liberia. Therefore, it is recommended that every patient with suspected malaria receive treatment for presumptive malaria when they first seek medical care at an Ebola treatment unit (ETU) or triage point (*9*,*10*). A diagnostic test to detect *Plasmodium* spp. parasitemia was implemented in the joint Centers for Disease Control and Prevention–National Institutes of Health (CDC–NIH) diagnostic laboratory located at the Eternal Love Winning Africa (ELWA) campus in Monrovia, Liberia.

*Plasmodium* spp. parasitemia can be detected by using rapid diagnostic tests (RDTs), light microscopy, or PCR. Of these methods, PCR is the most sensitive (0.004 parasites/μL) (*11*), and light microscopy is the reference diagnosis standard (5–10 parasites/μL). Both methods require highly qualified personnel and, in the case of PCR, specialized equipment to perform the test and analyze the results. An RDT produces results quickly and is simple to use, but it is the least sensitive method (>100 parasites/μL) (*12*), and most RDTs detect only *P. falciparum*. Light microscopy analysis poses difficulties in an EVD outbreak because of the required handling of infectious material and the need for proper personal protective equipment, so this diagnostic service usually is discontinued during EVD outbreaks. Therefore, because all blood samples submitted to the ELWA laboratory were analyzed by real-time quantitative reverse transcription PCR (qRT-PCR) for the presence of EBOV, we chose to screen for *Plasmodium* spp. parasitemia also by using qRT-PCR. Here we determine the feasibility of using molecular *Plasmodium* spp. diagnostics during an EVD outbreak and report the incidence of *Plasmodium* spp. parasitemia in persons with suspected EBOV infection.

## The Study

**Figure 1:**
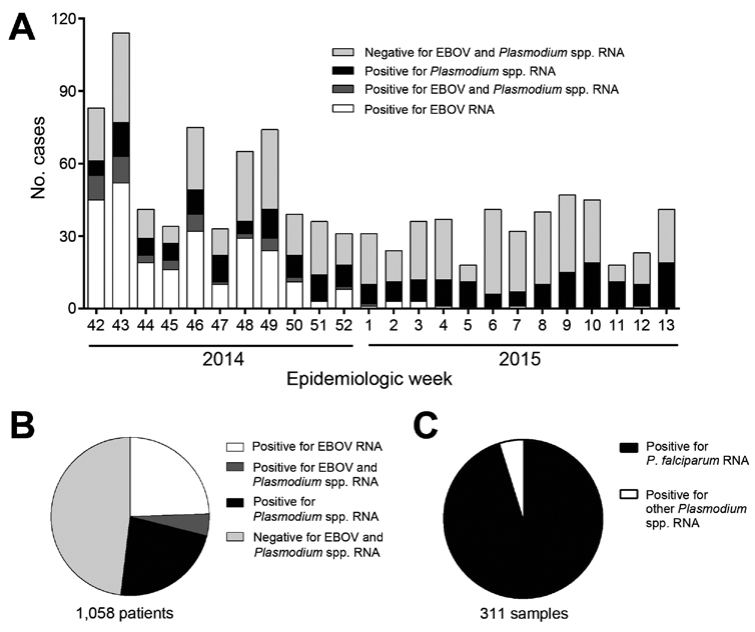
Prevalence of Ebola virus (EBOV) and *Plasmodium* spp. RNA in patient samples submitted to the Centers for Disease Control and Prevention–National Institutes of Health diagnostic laboratory at the Eternal Love Winning Africa campus in Monrovia, Liberia, from October 12, 2014 (epidemiologic week 42), through March 28, 2015 (week 13). Whole blood samples were inactivated, and RNA was extracted by using the QIAAmp Viral RNA Mini Kit (QIAGEN, Hilden, Germany). These samples were then tested for the presence of EBOV RNA and *Plasmodium* spp. RNA by real-time quantitative reverse transcription PCR (qRT-PCR) (*13*). A) Number of patients, as determined by qRT-PCR, positive for EBOV, *Plasmodium* spp., both, or neither (i.e., no EBOV and no *Plasmodium* spp.), by epidemiologic week. B) Total number of patients receiving a laboratory diagnosis of Ebola viremia, *Plasmodium* spp. parasitemia, both, or neither. C) A subset of 311 *Plasmodium* spp. qRT-PCR–positive samples that were retested with a qRT-PCR specific for *P. falciparum* (*14*).

During October 12, 2014–March 28, 2015, samples collected from 1,058 persons in Liberia with suspected EBOV infection were submitted to the CDC–NIH ELWA laboratory. The samples used for this research were collected for public health surveillance and not human subjects research, so institutional review board review and approval were not required. Early during the study period (October–November), most of the patients from whom samples were collected received a diagnosis of EBOV infection (Figure 1, panel A); toward the end, however, few cases of EBOV infection were diagnosed in Liberia. The number of patients who received a diagnosis of *Plasmodium* spp. parasitemia remained stable over time; thus, despite the lack of positive EBOV test results in the final months of the study period, overall, 40%–60% of patients each week received a diagnosis of EBOV infection, *Plasmodium* spp. parasitemia, or both (Figure 1, panel A). Of 1,058 samples tested, 259 (24.5%) were positive for EBOV alone, 243 (23%) were positive for *Plasmodium* spp. alone, and 47 (4.4%) were positive for both (Figure 1, panel B). Of 311 *Plasmodium*-positive samples that were further analyzed, 296 (95%) were positive for *P. falciparum* (Figure 1, panel C), confirming that *P. falciparum* was the main *Plasmodium* species causing parasitemia in our cohort.

**Figure 2:**
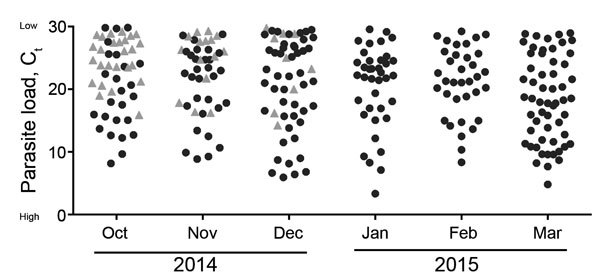
Inverse parasite load in patients with *Plasmodium* spp. parasitemia over time by month of sample submission, for samples submitted to the Centers for Disease Control and Prevention–National Institutes of Health diagnostic laboratory at the Eternal Love Winning Africa campus in Monrovia, Liberia, from October 12, 2014, through March 28, 2015. Cycle threshold (C_t_) values were detected by using real-time quantitative reverse transcription PCR. Triangles represent parasite loads in parasitemic patients co-infected with Ebola virus; circles represent patients with *Plasmodium* spp. parasitemia only. In West Africa, similar to other malaria-endemic regions, a large proportion of the population is infected with *Plasmodium* parasites without developing clinical disease. To compensate for this and the higher sensitivity of the PCR assay compared with light microscopy, we used a cutoff of C_t_ <30 rather than C_t_ <40 under the assumption that a 10-C_t_ difference would compensate for the ≈1,000-fold higher sensitivity of PCR over microscopy. This principle could be carried further to assume that a C_t_ <25 would be in the range detectable by the rapid diagnostic test. Of note, high C_t_ values correspond to low parasitemia levels and vice versa.

The cycle threshold (C_t_) values observed in the *Plasmodium* spp. qRT-PCR results are a proxy for parasite load (Figure 2). A high C_t_ value corresponds to a low-level parasitemia; the lower the C_t_ value, the higher the number of *Plasmodium* parasites detected. All patients in the cohort were triaged as having suspected EBOV infection, and thus all had clinical symptoms that might have been caused by EBOV, *Plasmodium* spp., or a different pathogen. Of note, significantly fewer *Plasmodium* parasites were detected in patients with EBOV infection than in patients who were not infected with EBOV (average *Plasmodium* C_t_ 24.7 vs. 20.37; p<0.01 by unpaired Student *t*-test), likely because clinical symptoms in these patients were caused by the EBOV infection rather than malaria. Some of the patients in our cohort with *Plasmodium* spp. parasitemia might have been asymptomatic carriers, especially those with a low-level parasitemia.

## Conclusions

One could argue that because all patients with febrile illness who were seen at many of the ETUs were given antimalarial treatment when they first sought medical care, providing laboratory testing for *Plasmodium* spp. parasitemia is not useful. Presumptive artemisinin-based combination treatment is recommended for all patients seen at ETUs, followed by prompt malaria diagnostic testing so that appropriate measures can be taken if oral treatment cannot be sustained because of clinical symptoms (*9*,*10*). Moreover, mathematical models predict a large increase in malaria in the epidemic region because of lapses in malaria control (*7*). Therefore, *Plasmodium* spp. parasitemia testing of all febrile patients seen at healthcare facilities could be used to determine whether this increase is indeed occurring so that countermeasures can be scaled up accordingly.

Differential diagnostic testing should be expanded to identify the cause of disease in the patients in our cohort whose samples tested negative for EBOV and *Plasmodium* spp., as was attempted in 1 laboratory in Sierra Leone (*15*). However, this kind of testing was difficult for several reasons. First, during the height of the EVD epidemic in Liberia, most laboratories were already working close to capacity while testing for EBOV alone. Testing for additional pathogens would have required the allocation of additional resources, including equipment and personnel. Second, it might not be possible to perform diagnostics using a whole blood sample for all the pathogens of differential diagnostic importance that can cause signs and symptoms similar to those of EVD (e.g., typhoid, bacterial sepsis, shigellosis, cholera, leptospirosis, dengue fever, rickettsioses, relapsing fever, meningitis, viral hepatitis, influenza, Lassa fever). Third, each of these pathogens likely is the cause of disease in only a small subset of febrile patients. Last, the prevalence of these pathogens likely differs in each outbreak region. Because broad-spectrum antimicrobial drugs and antimalarial drugs are routinely administered to all patients seen at an ETU, consideration for additional testing should focus on endemic pathogens that would not respond to these treatments.

By using PCR-based detection for *Plasmodium* spp. parasitemia, the need for additional handling of clinical specimens possibly infected with EBOV was eliminated. Thus, the addition of PCR-based diagnostic tests to detect *Plasmodium* spp. does not pose an additional safety risk to laboratory staff. Also, it is less time-consuming to add additional PCR reactions to a PCR run than to separately perform microscopy or an RDT on each sample, and these additions would add only ≈15 minutes to the overall time needed from sample submission to reporting of results. Moreover, if a multiplex approach is used, the additions would not require extra time or reagents. Taken together, our findings suggest that PCR-based testing for *Plasmodium* spp. parasitemia can be implemented easily and safely in laboratories performing EBOV diagnostics to assist with case-patient management during EVD outbreaks in malaria-endemic areas.
